# Retraction: Bipolar Affective Disorder: A Case of Antidepressant-Induced Hypomania

**DOI:** 10.7759/cureus.r20

**Published:** 2021-01-04

**Authors:** Zain Rizvi, S. M. Rafey Abidi, Anas Rafique, Aaesha Javed Sultan

**Affiliations:** 1 Internal Medicine, Shifa International Hospital, Islamabad, PAK; 2 Internal Medicine, Services Hospital, Lahore, PAK; 3 Psychiatry, Punjab Institute of Mental Health, Lahore, PAK; 4 General Surgery, Shifa International Hospital, Islamabad, PAK

This article was erroneously approved due to a technical error on the editorial side. The Cureus Journal of Medical Science greatly regrets this errors and any inconvenience caused to the authors or readers as a result. Following careful consideration by the journal's editorial board, we have determined that this article must be retracted as we have several critical concerns that cannot be addressed via erratum. These issues are listed below.

1. The dosage information for valproate, citalopram, quetiapine and valproate is not included nor is there sufficient explanation regarding the frequency and duration of treatment.
2. Information regarding sedation such as dose, frequency, protocol is not included.
3. The hypomania timeline and symptoms are not included nor is it explained why or how the hypomanic patient was admitted.
4. Details regarding the cessation of sedatives are not included.

